# Supplementary material for: Recombinant expression and preliminary characterization of Peptidyl-prolyl cis/trans-isomerase Rrd1 from Saccharomyces cerevisiae
Source: PLoS One. 2023 Jun 13;18(6):e0282749. doi: 10.1371/journal.pone.0282749 (PMC10263354; doi:10.1371/journal.pone.0282749)
Supplement: S1 File — (DOCX) [file pone.0282749.s001.docx]

**Supporting Information**

**Supplementary Figures:**

**Figures-S1**

**Figure-S1. Conserved domain analysis of recombinant Rrd1 protein.** Rrd1 protein belongs to PTPA-like protein superfamily.PPIases accelerate the folding of proteins. It catalyzes the cis-trans isomerization of proline imidic peptide bonds in oligopeptides. Acts as a regulatory subunit for serine/threonine-protein phosphatase 2A (PP2A) modulating its activity and is involved in the advancment of the G1 phase, DNA repair, dynamics of microtubule and bud morphogenesis.

**Figure-S2**

**
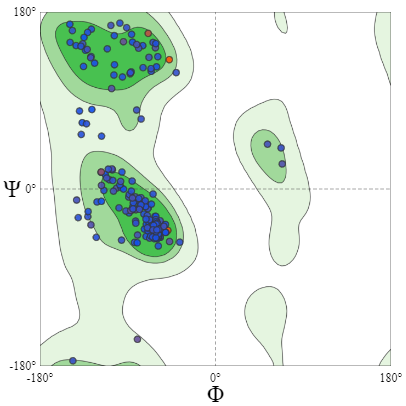
**

**Figure-S2.Ramachandran plot of Rrd1.** The Ramachandran plot represents the phi-psi torsion angles for all residues present in the proteinstructure of Rrd1. The coloring/shading on the plot showed various regions as the darkest areas (blue dots) indicate the 'core' regions showing the most favorable combinations of phi-psi angles and shows fair conservation in Rrd1.

**Figure-S3.**

**Figure S3.Cluster dendogram ofRrd1-like proteins from four distinct species were compared using the webPIPSA service.** Protein from species with extremely similar electrostatic potentials, including *Saccharomyces cerevisiae* (2IXN), *S. aureus* (3ROF) and *Homo sapiens*(2IXM), are found in one distnict subcluster, while*Escherichia coli*(1TE2) is found in a separate second subcluster.

**S1_raw_images**

**Author's contribution**

MK conceived the idea. MK performed the experiments. MA**^2^**, BK and RAL analyzed the data. MA^4^, MIK, AAA and AA helps in paper writing. AAA, MSA and MK have given critical inputs in the analysis and editing of the manuscript. All authors have read and approved the manuscript.

**Acknowledgment**

I greatly acknowledged my supervisor, Dr. Mohd. Sohail Akhtar for his guidance and support. Ahad Amer Alsaiari, Assistant professor, College of Applied Medical science, Department of Clinical Laboratories Science, Taif university, Saudi Arabia greatly acknowledged for professional grammar and revising the language editing in this manuscript. BK acknowledged Academy of Scientific and Innovative Research (AcSIR), Ghaziabad, 201002, India.

**Funding**

The Deanship of Scientific Research (DSR) at King Abdul Aziz University, Jeddah, Saudi Arabia has funded this project, under grant no. (KEP-25-130-42), which is greatly acknowledged.

**Declaration of competing interest**

The authors declare that they have no competing interests.
