# Supplementary material for: Recombinant expression and preliminary characterization of Peptidyl-prolyl cis/trans-isomerase Rrd1 from Saccharomyces cerevisiae
Source: PLoS One. 2023 Jun 13;18(6):e0282749. doi: 10.1371/journal.pone.0282749 (PMC10263354; doi:10.1371/journal.pone.0282749)
Supplement: S1 Raw images — (PDF) [file pone.0282749.s002.pdf]

# Figure-2

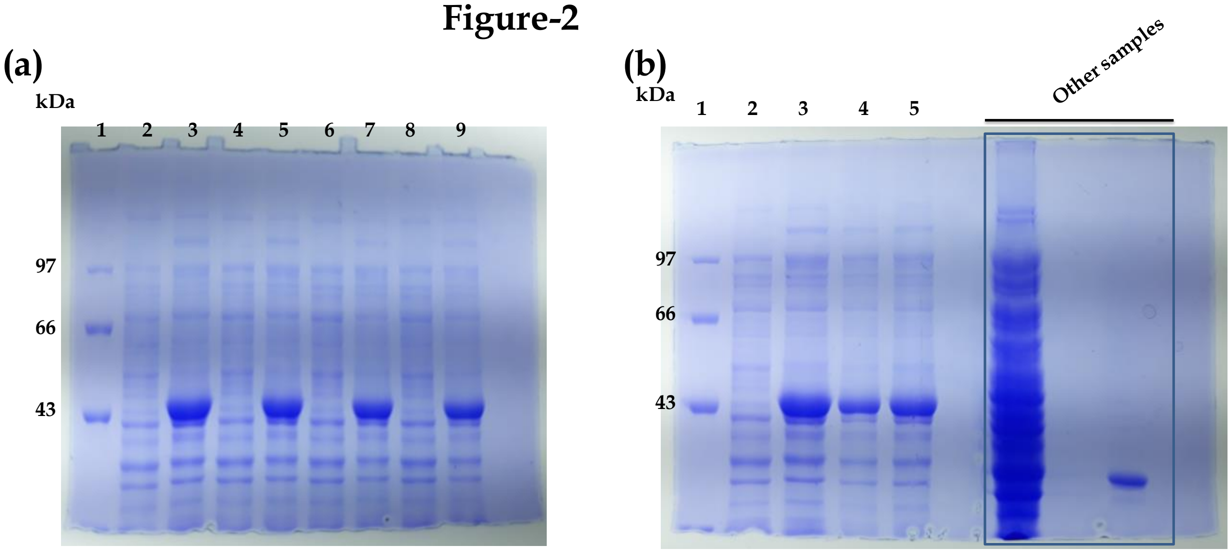


(a)

Lane1_ Molecular weight Marker
Lane2_ Uninduced C41
Lane3_ Induced C41
Lane4_ Uninduced BL-21
Lane5_ Induced BL-21
Lane6_ Uninduced BL21-CodonPlus
Lane7_ Induced BL21-CodonPlus
Lane8_ Uninduced Tuner
Lane9_ Induced Tuner

(b)

Lane1_ Molecular weight Marker
Lane2_ Uninduced
Lane3_ Induced
Lane4_Soluble fraction
Lane5_Insoluble fraction

# Figure-3

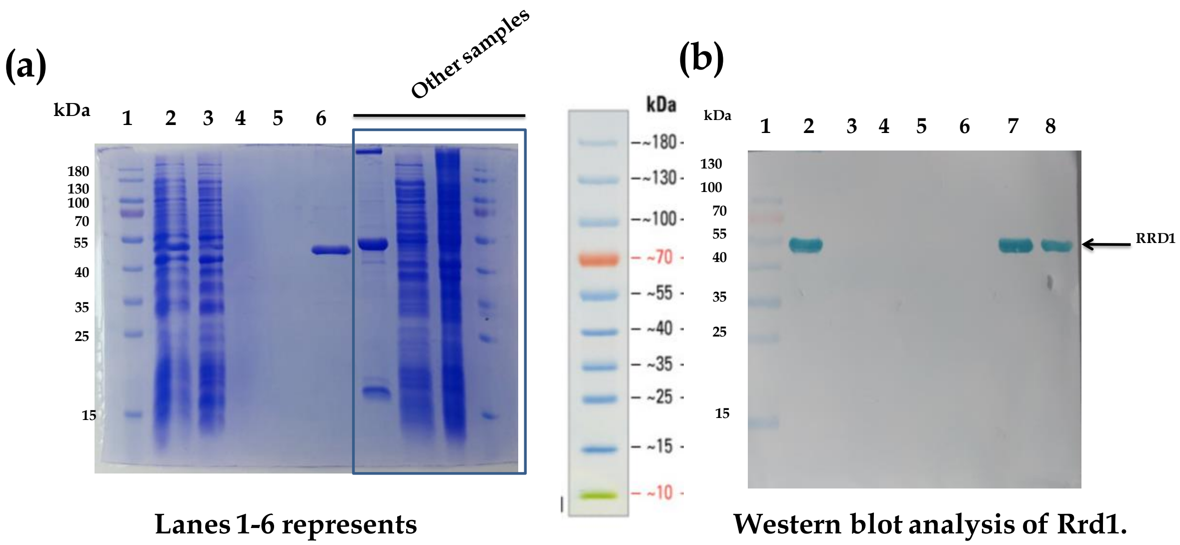


**Lanes 1-6 represents protein marker, lysate, sample after sonication, flow through, wash buffer1, wash buffer2 and eluted fraction from Ni NTA column respectively**

**Western blot analysis of Rrd1. Lanes 1-8 represents protein marker, lysate, sample after sonication, flow through, wash buffer1, wash buffer2 eluted fraction 1 and eluted fraction 2 from Ni NTA column respectively**
