# Supplementary material for: Recombinant expression and preliminary characterization of Peptidyl-prolyl cis/trans-isomerase Rrd1 from Saccharomyces cerevisiae
Source: PLoS One. 2023 Jun 13;18(6):e0282749. doi: 10.1371/journal.pone.0282749 (PMC10263354; doi:10.1371/journal.pone.0282749)
Supplement: Attachment — Submitted filename: Response to Reviewers.docx [file pone.0282749.s003.docx]

**Reviewer 1 comments:**

**Comment 1**. The authors conclude that the production of the recombinant protein can be used to obtain its biophysical characterization and to identify novel-associatin partners of Rrd1 protein. I believe, as the authors say, that the production of the recombinant protein can be used to obtain its biophysical characterization and to identify novel-associatin partners of Rrd1 protein, but in my opinion the work should be improved.

**Response:** We have tried our best to improve the language of the manuscript, as suggested by the reviewer. The suggestion has been incorporated in respective section of the data.

**Comment 2**. I would like to suggest that the authors should indicate the yield of purification. I would suggest that the authors try to eliminate many repetitive parts in Discussion and Conclusion paragraphs. I suggest a revision of the English language.

**Response:** Yield of purification have been incorporated in manuscript. We have tried our best to improve the language of the manuscript, as suggested by the reviewer. The suggestion has been incorporated in respective section of the data.

**Comment 3**. Is the ampicillin concentration (50mg/mL) correct? May be 50 micrograms/milliliter.

**Response:** We have tried our best to improve as suggested by the reviewer. The suggestion has been incorporated in respective section of the data.. Surely, we will take care of your valuable suggestions.

**Comment 4.** The resolution of the graphics is very bad. (Figure 1; Figure 3, C and D; Figure S1).

**Response:** We have improvised the respective figures as suggested by the reviewer. The suggestion has been incorporated in respective section of the data.

**Comment 5** In the “Overexpression and purification of the recombinant Rrd1 protein” paragraph, the parenthesis is missing (line 3).

**Response:** We have tried our best to improve the language and parenthesis of the manuscript, as suggested by the reviewer. The suggestion has been incorporated in respective section of the data.

**Comment 6** In the caption of Figure 2, opri,ization should be corrected.

**Response:** Thanks for valuable suggestions. Corrections have been incorporated.

**Comment 7** The caption of Figure 3, should be corrected.

**Response:** The caption of Figure 3, have been corrected as suggested by the reviewer.

**Comment 8** The column flow is once declared to be 0.3 and once more 0.4 mL/min.

**Response:** Thanks for valuable suggestions. The column flow rate has been corrected as suggested by the reviewer.

**Comment 9** The caption of Figure 4 and Figure 5 are not in italic.

**Response:** Thanks for valuable suggestions. Corrections have been incorporated.

**Comment 10** The font of the Figure 5 caption’s is different.

**Response:** The font of the Figure 5 caption’s is corrected as suggested by reviewer.

**Comment 11** The numerical sequence of the figures is wrong.

**Response:** The numerical sequence of the figures is corrected. Corrections have been incorporated.

**Reviewer 2 comments:**

**Comment** 1 The manuscript by Mohd Kashif et al. entitled “Recombinant expression and preliminary characterization of Peptidyl-prоlyl cis/transisоmerase Rrd1 from Saccharomyces cerevisiae” report cloning, expression and purification of the Rrd1 from Saccharomyces cerevisiae, and also an initial characterization. The authors declare that the protein is correctly cloned, expressed in the E. Coli strain, and purified. The authors, by the CD and the Spectro fluorescence, declare also that the protein is correctly folded. The aim of this work is interesting (and this reviewer appreciates it), the paper is not well done in almost all parts, and the manuscript in the present form demands a deep revision before it can be published, so this reviewer suggests a major revision of the paper.

**Response:** Thanks for valuable suggestions. We have tried our best to improve the language and majorly revised the manuscript, as suggested by the reviewers. The suggestion has been incorporated in respective section of the data.

**Comment 2:** The abstract needs to be revised according to the following suggestions.

**Response:** Abstract has been revised as suggested by the reviewers. The suggestion has been incorporated in abstract section.

**Comment 3:** The introduction section is too long and dispersive; nothing is reported on the aim of the paper. The results and the methods used should be removed from this section.

**Response:** Thanks for valuable suggestions. Corrections have been incorporated.

**Comment 4:** The section Materials and Methods describe also the results obtained. In this way is difficult to read and understand the paper, please split this section into two sections (e.g. M&M and Results).

**Response:** Thanks for valuable suggestions. We have separated M&M and Results section into two sections. Corrections have been incorporated.

**Comment 5:** About the purification is not reported the yield per gram of humid pellet, is not reported a spectroscopic analysis for the purity evaluation of the protein and for the quantization of the protein.

**Response:** Table explaining yield of purification have been incorporated in manuscript. The suggestion has been incorporated in respective section of the data.

**Comment 6:** In the paragraph “Optimization of recombinant Rrd1 expression and solubility” the most important info is not reported “data not show”, why?

**Response:** Thanks for valuable suggestions. Corrections have been incorporated.

**Comment 7:** Some parts of the computational analysis should be reported before the expression and purification and the rest before the characterization.

**Response:** Thanks for valuable suggestions. Corrections have been incorporated.

**Comment 8:** In the paragraph “Primary sequence analysis and homology modeling of Rrd1” is reported (fig 3a) but it does not match with the text.

**Response:** Thanks for valuable suggestions. Corrections have been incorporated in respective area.

**Comment 9:** The paragraph “Structure analysis of Rrd1 protein” should be completely revised or removed. The CD spectra analysis is too simplistic, no spectra post-analysis was done. An investigation in the near-UV should be performed. By the fluorescence analysis performed (just steady-state spectra) is not possible to define the tertiary structure or the right folding of the protein, a deeper fluorescence analysis should be performed to declare the correct folding.

**Response:** The paragraph “Structure analysis of Rrd1 protein” now has been completely revised as suggested. Surely, we will take care of your valuable suggestions, whenever we shall plan such type of studies in future. Thanks for suggestions.

**Comment 10:** As a consequence of the point before also the Discussion and the Conclusions sections need to be revised.

**Response:** Discussion and the Conclusions sections has been revised as suggested. Thanks for valuable suggestions

**Comment 11:** Please revise the text for some misspellings.

**Response:** revised as suggested. Thanks for valuable suggestions

**Comment 12:** Figure 3 panels c and d are in low resolution, please improve.

**Response:** Figure 3 panels c and d have been improved.
**Comment 14:** Figure S1 is in low resolution, please improve it.

**Response:** Figure S1 is in low resolution has been improved
